# The Morbidity of Reoperative Surgery for Recurrent Benign Nodular Goitre: Impact of Previous Unilateral Thyroid Lobectomy versus Subtotal Thyroidectomy

**DOI:** 10.1155/2014/231857

**Published:** 2014-01-19

**Authors:** Navin Rudolph, Claudia Dominguez, Anthony Beaulieu, Pierre De Wailly, Jean-Louis Kraimps

**Affiliations:** Department of Endocrine Surgery, University Hospital of Poitiers, 86021 Poitiers, France

## Abstract

*Background*. Subtotal thyroidectomy (STT) was previously considered the gold standard in the surgical management of multinodular goitre despite its propensity for recurrence. Our aim was to assess whether prior STT or unilateral lobectomy was associated with increased reoperative morbidity. *Methods*. A retrospective analysis was conducted extracting data from our endocrine surgical database for the period from January 1991 to June 2006. Two patient groups were defined: Group 1 consisted of patients with previous unilateral thyroid lobectomy; Group 2 had undergone previous STT. Specific outcomes investigated were transient and permanent recurrent laryngeal nerve (RLN) injury and hypoparathyroidism. *Results*. 494 reoperative cases were performed which consisted of 259 patients with previous unilateral lobectomy (Group 1) and 235 patients with previous subtotal thyroidectomy (Group 2). A statistically significant increase relating to previous STT was demonstrated in both permanent RLN injury (0.77% versus 3.4%, RR 4.38, *P* = 0.038) and permanent hypoparathyroidism (1.5% versus 5.1%, RR 3.14, *P* = 0.041). Transient nerve injury and hypocalcaemia incidence was comparable. *Conclusions*. Reoperative surgery following subtotal thyroidectomy is associated with a significantly increased risk of permanent recurrent laryngeal nerve injury and hypoparathyroidism when compared with previous unilateral thyroidectomy. Subtotal thyroidectomy should therefore no longer be recommended in the management of multinodular goitre.

## 1. Background

Subtotal thyroidectomy (STT) was for many years the accepted standard of management for benign multinodular goitre. Although its role has significantly diminished with the recognition of the safety of total thyroidectomy, it continues to be practised in several centres worldwide both in locations endemic and nonendemic for this disease. The purported benefits of the subtotal approach include a reduced morbidity profile with respect to recurrent laryngeal (RLN) injury and hypoparathyroidism as well as a reduced need for thyroid hormone replacement therapy. The exact deployment of the surgical technique has varied amongst institutions with regard to the size and location of the thyroid remnant and whether it has been performed unilaterally or bilaterally. This marked potential for heterogeneity has hampered reliable scientific appraisal of the technique's efficacy.

Despite these methodological encumbrances, several concerns regarding STT have emerged that challenge its legitimacy within the surgical armamentarium of the thyroid surgeon. First, recurrent disease, often manifesting many years following the initial surgery, is identified in a significant number of patients [1]. Moreover, thyroid insufficiency and consequent need for thyroid hormone replacement therapy are only infrequently eradicated [2]. The presence of a thyroid remnant is problematic if an incidental malignancy is discovered. Finally, the identification of recurrent disease heralds technically demanding reoperative surgery fraught with potential for significant morbidity.

Alongside this, total thyroidectomy (TT) has been demonstrated to be a safe and efficacious procedure. Technological advancements in haemostatic vessel sealing devices have hastened the operative technique significantly and more recently nerve monitoring has been widely adopted to further combat the low rates of RLN paralysis already demonstrated. Other benefits relate to its superiority in cases of malignancy by removing all gross thyroid and potentially malignant tissue, facilitating radioactive iodine (RAI) ablation therapy and facilitating surveillance with ultrasound imaging of the thyroid bed and thyroglobulin (Tg) monitoring.

Our unit is a high volume tertiary endocrine surgery centre performing over 500 thyroid procedures per year. Having previously practiced STT for many years we have a vast experience with recurrent benign thyroid goitre following this procedure. In addition, a large number of patients who underwent unilateral thyroid lobectomy for unilateral benign nodular disease have subsequently required completion totalization thyroidectomy for contralateral recurrent disease. Our unique study approach endeavoured to assess whether reoperative surgery in these two settings conferred any difference in morbidity specifically with regard to RLN injury and hypoparathyroidism.

## 2. Materials and Methods

A retrospective analysis was conducted utilizing our endocrine surgical database for the period from January 1991 to June 2006. There were 494 patients that required reoperation for recurrent benign goitre and were thus selected for this study. The indications for the reoperative surgery included enlarging neck lump, pressure symptoms, and imaging suspicious for malignancy. The patients were divided into two groups on the basis of the previous surgery: group 1 consisted of patients who had previous unilateral thyroid lobectomy; group 2 included patients who had undergone prior subtotal thyroidectomy. In all cases both the initial and reoperative procedure had been performed at our own institution.

The technique of unilateral extracapsular thyroidectomy at our institution has been well documented previously [3]. Our subtotal thyroidectomy procedure performed during this period is detailed here to avoid potential ambiguity. Careful preoperative study of the thyroid ultrasound was paramount in order to appreciate the location of the nodules within each thyroid lobe. A small (less than 5 grams) homogeneous remnant was left unilaterally at either the superior pole or posteriorly depending on the location of the sonographically or intraoperatively detected nodules. This combination of unilateral lobectomy and unilateral subtotal resection has been labelled elsewhere as the Dunhill procedure [4]. The operations were all performed by or under the direct supervision of a senior endocrine surgeon (JLK) following a highly standardized procedure. Postoperative nonsuppressive thyroxine therapy was employed for restoration of euthyroidism as dictated by thyroid function tests (thyroid-stimulating hormone (TSH); free T4 and T3).

Prior to reoperative surgery all patients underwent imaging by ultrasound; computed tomography scans and technetium thyroid uptake scans were performed on an individualized basis depending on the extent of the recurrence. Intraoperative nerve monitoring was used in all reoperative cases. Fibreoptic flexible laryngoscopy was routinely performed in all patients both preoperatively and on the first postoperative day permitting an accurate calculation of our temporary RLN injury rate. Patients with any detected abnormalities at this examination underwent a further laryngoscopy at 6 months postoperatively; vocal cord dysfunction at this stage was defined as permanent RLN injury. Calcium levels were obtained on the first and second postoperative days and at a 6-month followup appointment. Hypocalcaemia was defined as less than 2.00 mmol/L and permanent if requiring ongoing oral calcium supplementation beyond 3 months. Morbidity arising from the initial operation was not the intended focus of this study and was excluded from the analysis; only new morbidity events specifically relating to the reoperative surgery were included. Descriptive statistics were obtained and data subjected to analysis by Fisher's exact test and chi-square test to examine the relative risk of reoperative morbidity for group 1 and group 2. Statistical significance was accepted at *P* < 0.05.

## 3. Results

During the study period our unit performed thyroid surgery on 6780 patients. There were 494 patients that required reoperation for recurrent benign goitre, which constituted 7.3% of the unit's thyroid surgery throughput during this period.

Group 1 comprised 259 patients with previous thyroid lobectomy and group 2 comprised 235 patients with previous subtotal thyroidectomy (Table 1). The groups displayed demographic parity with respect to mean age (group 1, 38 years; group 2, 40 years) and female predominance (92% and 91%, resp.).

The mean interval between initial surgery and reoperation was 15.2 years in group 1 and 13.9 years in group 2. The indication for reoperation in groups 1 and 2 was also comparable: isolated nodules in 14.7% and 15.3% and multinodular goitre in 85.3% and 84.7%, respectively.

The impact of the initial surgery on the morbidity related to the reoperative case was statistically significant for both permanent RLN injury and permanent hypocalcaemia.

Permanent RLN palsy was observed in only 2 from group 1 and 8 from group 2 (Table 2). This correlates with a statistically significant detrimental effect of initial subtotal thyroidectomy on long-term RLN function (*P* < 0.038). This indicates a relative risk increase of 4.38 in patients who underwent initial subtotal thyroidectomy (CI_95_ 0.94–20.4). Transient paralysis was observed in both groups (group 1, 6.18%; group 2, 5.53%). The majority of patients in both groups, however, have no disturbance in postoperative RLN function (group 1, 83.9%; group 2, 83.4%).

Permanent hypocalcaemia was observed in 1.54% of group 1 patients and 5.11% of group 2 patients (Table 3). Again, this reflected a statistically significant detrimental effect of initial subtotal thyroidectomy on the development of permanent hypocalcaemia following reoperative surgery (*P* < 0.041) and correlates with an relative risk increase of 3.14 (CI_95_ 1.09–9.59). No association was determined for temporary hypocalcaemia and the nature of prior surgery (RR 0.69, CI_95_ 0.41–1.14). Of note, postoperative normocalcaemia was evident in 84.9% and 85.9% of patients from group 1 and group 2, respectively.

Incidental malignancy within the reoperative specimen was determined in 21 patients (4.22%). There was an equitable distribution within the two groups, with 11 cases in group 1 and 10 cases in group 2.

## 4. Discussion

The optimum extent of initial surgery in the management of benign thyroid goitre continues to generate considerable controversy. The debate between the safety and efficacy of a total versus a less than total thyroidectomy has successfully accomplished the widespread adoption of total thyroidectomy although not fully extinguishing the practice of subtotal thyroidectomy in several centres worldwide. Our study, drawing on a vast experience with reoperative surgery in benign thyroid disease, approaches the debate from a different angle. Rather than focussing on the morbidity of an initial subtotal thyroidectomy versus an extracapsular technique, our study represents the first direct comparison on the morbidity relating to the reoperative surgery in the setting of previous subtotal and unilateral thyroid resections. Although the results may appear intuitive, the study did expose some interesting and important facets of reoperative surgery in these circumstances.

Reoperative thyroid surgery is inherently difficult on account of the distortion of central neck area anatomy and fibrotic encasement of important structures such as the recurrent laryngeal nerve [5]. This has led to the recommendation by several authors to avoid reoperations by performing definitive initial treatment [6]. Despite these difficulties Levin et al. demonstrated that reoperations could still be performed with minimal morbidity [7]. In their series, a low permanent RLN injury rate of less than 1% and permanent hypoparathyroidism rate of 3.8% was attained—the authors consequently stressed that, for patients manifesting with recurrent disease, reoperative surgery should not be withheld for fear of generating the aforementioned complications. Our rates of permanent RLN palsy (2%) and hypoparathyroidism (3.2%) from the two groups combined likewise demonstrate that satisfactory outcomes are achievable within specialized centres. Other authors have published series of reoperative cases with permanent RLN rates of 0–1.5% and highlighted that, although being hardly an innocuous procedure, reoperative surgery is safe in the hands of experienced surgeons; however, a complete initial procedure should obviate the exposure to this unnecessary additional risk [8, 9].

A different scenario exists when a patient requires secondary thyroid surgery for recurrent benign disease with a background of unilateral hemithyroidectomy. In this situation, where the contralateral side is completely untouched, no increased risk is conferred as shown by Chao et al. and confirmed in our study with rates of permanent RLN injury and permanent hypoparathyroidism rates of 0.77% and 1.54%, respectively [10]. However, previous STT, in which both sides have been dissected, is associated with an up to fivefold increase in complications with reoperative surgery [11, 12]. Despite the scar tissue and degenerative changes cited by Katz and Bronson as the principle culprit factors relating to reoperative morbidity [13], Bron and O'Brien found no significant correlation between complication rate and previous surgery [14]. Transient hypoparathyroidism was seen in 13.5% of group 1 patients in our study representing a nonstatistically significant difference from the previous STT group. Germane to this finding, Barczyński et al. remark that transient hypoparathyroidism following TT in an era where parathyroid autotransplantation is common should be viewed as a sequel rather than a complication [15].

The technique of reoperative thyroid surgery is clearly important when analysing complications. Farrag and colleagues have recently promulgated an algorithm for safe and effective thyroid bed surgery for malignancy although many aspects can be readily extrapolated to the benign sphere as well [5]. The principle tenets of the algorithm include preoperative high-resolution ultrasound examination, preoperative vocal fold examination by fibreoptic laryngoscopy, and routine nerve identification that should be facilitated by the use of intraoperative nerve monitoring (IONM). Several of these attributes are endorsed by international thyroid surgery guidelines [16]. Additionally, Menegaux et al. recommend a lateral approach to the thyroid bed with division of the infrahyoid musculature in an effort to avoid fibrous tissue surrounding the thyroid remnant [17].

TT emerged as an alternative to STT initially in the malignant domain. Clark embraced the technique for management of well-differentiated thyroid cancer and demonstrated the safety of the technique and low complication rate [18]. It is recommended as the operation of choice in all current treatment guidelines for thyroid cancer [19]. The purported benefits of a total gland resection in thyroid cancer include the removal of the primary tumour, elimination of any potential contralateral disease and facilitation of postoperative RAI ablation, Tg surveillance, and ultrasound scanning of the thyroid bed [20]. These benefits are not present when dealing with benign disease. Proponents of a subtotal resection claim a reduced rate of RLN injury and hypoparathyroidism and assert that the majority of thyroid malignancies detected fortuitously on final histopathology are of limited clinical significance. Furthermore, if recurrences do occur they can be managed surgically when indicated with less morbidity than if total thyroidectomy was performed on all cases of thyroid malignancy at the outset [20].

Numerous publications have since established the low morbidity of the extracapsular TT procedure. Mishra et al. demonstrated an 0.8% permanent RLN rate and 1.6% permanent hypoparathyroidism rate whilst Müller et al. found a 0.9% rate for both morbidities [21, 22]. Many other studies confirmed similar findings [23–25]. Serpell and Phan found a permanent RLN palsy rate of 0.3% and hypoparathyroidism rate of 1.8% and concluded that TT can be performed safely in a standard endocrine surgical unit with low complication rates matching world centres of excellence [26]. Documented high rates of incidental thyroid malignancy fortified the growing argument in favour of total thyroidectomy. Our present study revealed an incidental papillary microcarcinoma rate of 4.22% that was equally distributed within the two groups. We have previously published a 3% rate of occult carcinoma, somewhat lower than other subsequently published series from Bron and O'Brien (4.6%) [14], Colak et al. (7.4%) [27], and Levin et al. (22%) [7]. Menegaux et al. determined that thyroid cancer might be found in approximately 10% of reoperative cases for recurrent goitre even though the preceding operation was performed for benign disease [28]. By the same token, Tezelman et al. found that within a cohort of patients diagnosed with thyroid cancer following a subtotal thyroidectomy, completion resection of the thyroid remnants yielded papillary microcarcinoma in 5.26% [1].

Recurrence of goitre and failure to prevent hypothyroidism have further weakened the validity of subtotal thyroidectomy as a surgical strategy for benign thyroid disease. Goitre recurrence rates associated with STT range from 7.1% to 43% [1, 12]. The incidence of recurrence appears directly related to the length of surveillance [29, 30]—although our study and others have found that a peak incidence of recurrence occurs at approximately 13 to 15 years from the primary operation [31], a 43% recurrence rate may be observed with up to 30 years of followup [12]. Many have noted the failure of thyroxine suppression to prevent recurrence with a 14.5% recurrence rate demonstrated in the study by Pappalardo et al. in spite of drug prophylaxis [24]. Conversely, thyroxine replacement is not obviated in 36.6–47.8% of subtotal thyroidectomy procedures and hence should not be used to justify practice of the procedure [32].

The fundamental difficulty with STT is allotting a tissue remnant unaffected by the nodular process. Colak et al. highlighted the predicament of leaving healthy tissue intact in patients with huge goitres where the nodular disease often reaches the dorsal capsule [27]. There is generally an increasing recognition that the nodular transformations in multinodular goitre inherently encompass the entire gland; hence, although STT permits a reduction in the bulk of disease, it is not an optimal treatment [33]. Moreover, the remnant posterolateral tissue often extends into the retrotracheal and retrooesophageal areas where recurrence portends early pressure symptoms that may require technically demanding reoperative surgery [34]. We have previously shown that multinodular goitre is a highly significant risk factor for recurrence in benign thyroid disease where a less than total thyroidectomy has been performed [35]. Recently, a novel study by Tekin et al. demonstrated that *Ki-67* proliferation marker levels in remnant STT thyroid tissue were significantly higher than in normal thyroid tissue [36]. They found that despite the relatively small size of remnant micronodules the high *Ki-67* levels reflect high cellular mitotic activity and ensuing significant goitrogenic potential of remnant tissue. Furthermore, the existence of micronodules in the remnant specimens harbouring similar proliferation index values as the main thyroid specimen establishes the homogeneous nature of the parenchymal alteration in multinodular goitre. Gerard et al. postulate that recurrent goitres resistant to thyroxine suppression may arise due to the polyclonal nature of nodule formation involving goitrogenic insulin-like growth factors and their binding proteins. These may occur separate to iodine deficiency related mechanisms of goitre development based on TSH and vascular-endothelial growth factor (VEGF) angiogenesis [37].

Finally, the often-overlooked denominator and perhaps most crucial determinant of morbidity is surgical technique. Thomusch et al. commented on the importance of “well-trained surgeons using an appropriate intraoperative technique” in the performance of total thyroidectomy [38]. The ability to perform a safe total thyroidectomy is clearly a direct derivative of one's surgical training and experience [39]. The evolution of more dedicated endocrine surgery training programmes and specialized units combined with technological refinements such as IONM is likely to further enhance the safe implementation of TT for both malignant and benign thyroid disease alike [40].

## 5. Conclusion

Reoperative surgery for recurrent benign thyroid disease is associated with increased morbidity when preceded by initial subtotal thyroidectomy. Associated high levels of recurrence and increased permanent RLN injury and hypoparathyroidism rates seen in this setting call for the abandonment of this procedure in favour of total thyroidectomy. It should be noted however that successful reoperative thyroid surgery performed by experienced, well-trained surgeons may be accomplished with low overall rates of morbidity.

## Figures and Tables

**Table 1:** Group demographics and timing and indication for reoperation.

	Group 1 *n* = 259	Group 2 *n* = 235
Sex		
Male	21 (8%)	20 (9%)
Female	238 (92%)	215 (91%)
Age		
Age at first operation	38.0 years	40.0 years
Age at reoperation	53.2 years	53.9 years
Interval between initial and reoperative surgery	15.2 years	13.9 years
Indication for reoperation		
Isolated nodule	38 (14.7%)	36 (15.3%)
Multinodular goitre	221 (85.3%)	199 (84.7%)

**Table 2:** Incidence of RLN injury following reoperative surgery.

	Group 1 (lobectomy) *n* = 259	Group 2 (subtotal) *n* = 235	*P* value	Relative risk
Normal laryngoscopy	241 (83.9%)	214 (83.4%)		
Transient RLN paralysis	16 (6.18%)	13 (5.53%)	0.85	0.92
Permanent RLN paralysis	2 (0.77%)	8 (3.4%)	0.038	4.38

**Table 3:** Incidence of hypocalcaemia following reoperative surgery.

	Group 1 (lobectomy) *n* = 259	Group 2 (subtotal) *n* = 235	*P* value	Relative risk
Normocalcaemia	220 (84.9%)	202 (85.9%)		
Temporary hypocalcaemia	35 (13.5%)	21 (8.93%)	0.15	0.69
Permanent hypocalcaemia	4 (1.54%)	12 (5.1%)	0.041	3.14
